# *Porphyromonas gingivalis*-Induced Cognitive Impairment Is Associated With Gut Dysbiosis, Neuroinflammation, and Glymphatic Dysfunction

**DOI:** 10.3389/fcimb.2021.755925

**Published:** 2021-12-01

**Authors:** Li Chi, Xiao Cheng, Lishan Lin, Tao Yang, Jianbo Sun, Yiwei Feng, Fengyin Liang, Zhong Pei, Wei Teng

**Affiliations:** ^1^ Hospital of Stomatology, Guangdong Provincial Key Laboratory of Stomatology, Institute of Stomatological Research, Guanghua School of Stomatology, Sun Yat-sen University, Guangzhou, China; ^2^ Department of Neurology, The First Affiliated Hospital, Sun Yat-sen University, Guangzhou, China; ^3^ Guangdong Provincial Key Laboratory of Diagnosis and Treatment of Major Neurological Diseases, National Key Clinical Department and Key Discipline of Neurology, Guangzhou, China; ^4^ Department of Neurology, Huashan Hospital, Fudan University, Shanghai, China

**Keywords:** *Porphyromonas gingivalis*, cognitive impairment, neuroinflammation, glymphatic system, gut–brain axis

## Abstract

**Background:**

Periodontal pathogen and gut microbiota are closely associated with the pathogenesis of Alzheimer’s disease (AD). *Porphyromonas gingivalis* (Pg), the keystone periodontal pathogen, can induce cognitive impairment. The gut has a connection and communication with the brain, which is an important aspect of the gut–brain axis (GBA). In the present study, we investigate whether Pg induces cognitive impairment through disturbing the GBA.

**Methods:**

In this study, Pg was orally administered to mice, three times a week for 1 month. The effects of Pg administration on the gut and brain were evaluated through behaviors, gut microbiota, immune cells, glymphatic pathway clearance, and neuroinflammation.

**Results:**

Pg induced cognitive impairment and dysbiosis of gut microbiota. The α-diversity parameters did not show significant change after Pg administration. The β-diversity demonstrated that the gut microbiota compositions were different between the Pg-administered and control groups. At the species level, the Pg group displayed a lower abundance of *Parabacteroides gordonii* and *Ruminococcus callidus* than the control group, but a higher abundance of *Mucispirillum schaedleri*. The proportions of lymphocytes in the periphery and myeloid cells infiltrating the brain were increased in Pg-treated animals. In addition, the solute clearance efficiency of the glymphatic system decreased. Neurons in the hippocampus and cortex regions were reduced in mice treated with Pg. Microglia, astrocytes, and apoptotic cells were increased. Furthermore, amyloid plaque appeared in the hippocampus and cortex regions in Pg-treated mice.

**Conclusions:**

These findings indicate that Pg may play an important role in gut dysbiosis, neuroinflammation, and glymphatic system impairment, which may in turn lead to cognitive impairment.

**Graphical Abstract:**
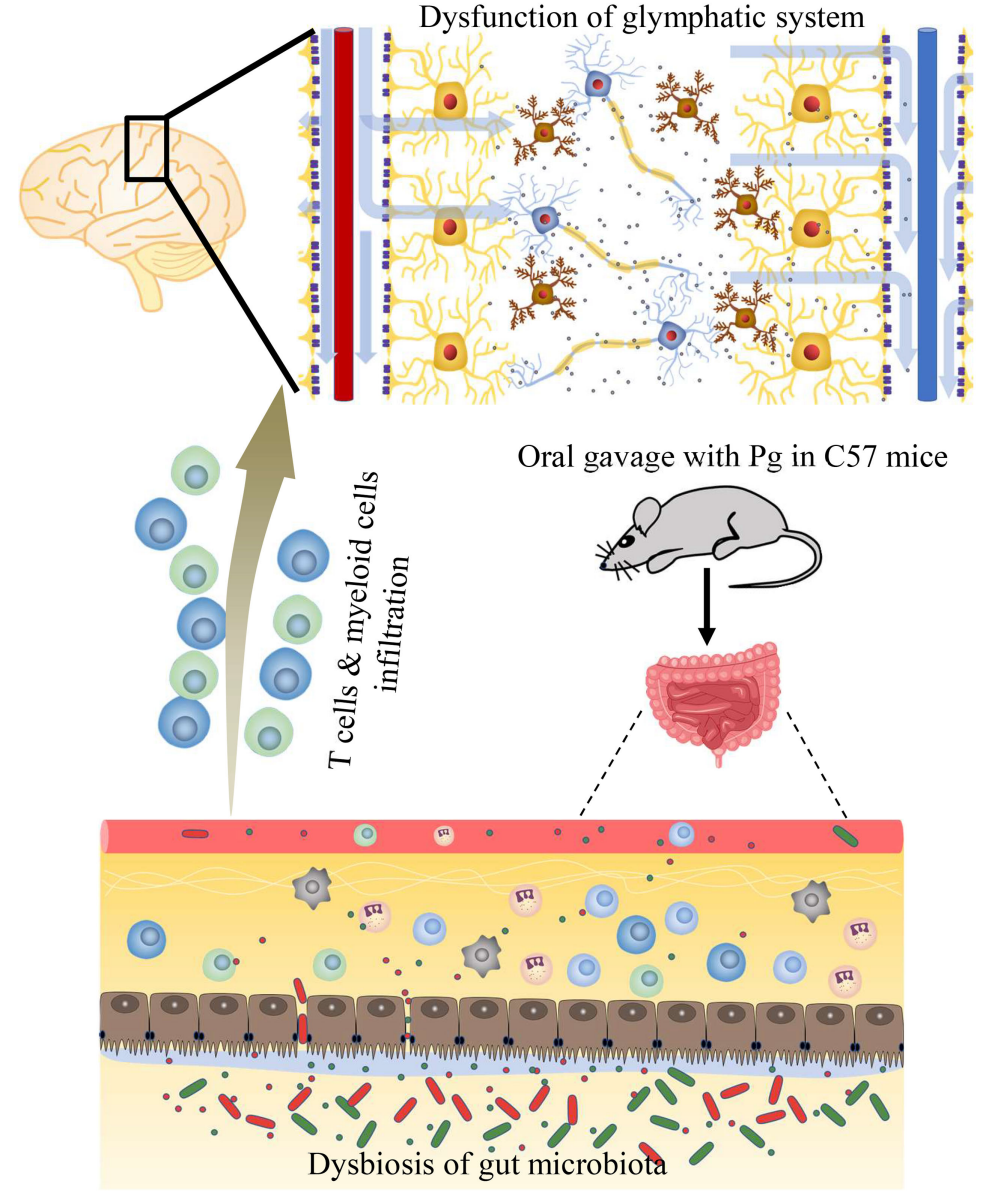
The periodontal pathogen *P. gingivalis* induces cognitive impairment by disturbing gut-brain axis.

## Introduction

*Porphyromonas gingivalis* (Pg) is a keystone pathogen in periodontitis (Yang et al., 2019). In addition to its oral effect, Pg is closely related with the occurrence and development of numerous systemic diseases, such as atherosclerosis (Xie et al., 2020), diabetes (Tian et al., 2020), and Alzheimer’s disease (AD) (Diaz-Zuniga et al., 2020). Cognitive impairment is an early symptom of AD and Pg was found to be closely related to cognitive impairment (Noble et al., 2009; Zhang et al., 2018). Amyloid plaques are aggregation of beta-amyloid peptides (Aβ) that accumulate in the brain, damaging and destroying neurons and resulting in progressive cognitive impairment. It is reported that Pg can not only promote the Aβ deposit in the central system (Dominy et al., 2019), but also induce macrophages to produce Aβ, which may contribute to the central deposition (Nie et al., 2019). Virulence factors of Pg such as LPS and gingipain can induce inflammatory responses. Pg-LPS can induce neuronal inflammation through the TLR4/NF-κB pathway (Zhang et al., 2018). Gingipain induces the migration of microglia to the site of infection and leads to neuroinflammation (Nonaka and Nakanishi, 2020). Inhibitors of Pg virulent factors could ameliorate infection and reduce amyloid plaque production and neuroinflammation (Dominy et al., 2019).

Pg of oral origin can induce the dysbiosis of gut microbiota (Kato et al., 2018; Ohtsu et al., 2019). The oral–gut connection of Pg occurs during common activities such as chewing and swallowing. Gut microbiota composition plays a part in the regulation of brain functions, including social behavior, motor dysfunction, and cognitive functions *via* the gut–brain axis (GBA) (Erny et al., 2015; Rogers et al., 2016; Sampson et al., 2016; Cryan et al., 2020). The GBA is regarded as a bidirectional connection between the central nervous system (CNS) and the gastrointestinal tract of the body. It contains various direct and indirect pathways between the cognitive center in the brain and peripheral intestinal function. Regulation of the GBA is critical for maintaining homeostasis, including that of the CNS. The regulatory effects of gut microbiota on the brain can be mediated by the immune aspect of the GBA (van Sadelhoff et al., 2019). Peripheral immune cells in brain parenchyma are maintained at a low level under normal condition. In the state of disease, infiltrated lymphocytes and myeloid cells often turn to damage CNS tissue (Gate et al., 2020; Dressman and Elyaman, 2021; Savinetti et al., 2021).

Neuroinflammation is a general characteristic of the CNS in neurological disorders and is considered as a potential factor of cognitive impairment (Gilhus and Deuschl, 2019). The neuroinflammatory responses, such as activation of gliocytes and expression of proinflammatory cytokines, could exacerbate the CNS microenvironment in diseases and may make a contribution to acceleration of cognitive impairment. Deposition of Aβ is considered as one of the pathological features of AD. In normal physiological conditions, Aβ production and clearance are maintained at a balanced level. In the past, the CNS is believed to be immune privileged, lacking a classic drainage of the lymphatic system. But now, as is known to all, the CNS goes through continuous immune surveillance (Louveau et al., 2015). The glymphatic system has a significant effect on the clearance of brain metabolic wastes (Abbott et al., 2018). The clearing efficiency of the glymphatic pathway can be influenced by sleep deprivation (Nedergaard and Goldman, 2020), some drugs, and neuroinflammation (Sundaram et al., 2019). The glymphatic pathway includes the perivascular space (PVS) influx of cerebrospinal fluid (CSF) into the brain interstitial fluid (ISF), followed by the clearance of ISF along draining veins (Iliff et al., 2012). The continuous movement of fluid through the interchange between the CSF and ISF is critical to clear interstitial solutes. Dysfunction of the glymphatic pathway leads to metabolic waste accumulation, such as Aβ, which is considered to contribute to AD (Arbel-Ornath et al., 2013; Plog and Nedergaard, 2018).

Therefore, we hypothesized that Pg might induce cognitive impairment through regulating the GBA in middle-aged mice.

## Method

### Animals

All experiments were approved by the Institutional Animal Care and Use Committee, Sun Yat-Sen University (Guangzhou, China; approval no. 000439). In this study, 9- to 10-month-old male C57BL/6J mice were acquired from Vital River (Beijing, China). All animals were raised in a specific pathogen-free facility of Sun Yat-Sen University, with ad libitum food and water. All animals were randomly assigned to two groups: control and Pg group (*n* = 15).

### Oral Administration of Pg

Mice were given by oral gavage 10^9^ colony-forming units (CFU) of Pg in total, and the Pg was resuspended in 0.1 ml phosphate buffered saline (PBS) with a concentration of 2% carboxymethyl cellulose (CMC) (Sigma Aldrich, St. Louis, MO, USA). This suspension was given three times a week for 4 weeks. The control group was given a suspension without Pg.

### Morris Water Maze Test

The Morris water maze (MWM) test was conducted, based on the protocol previously described (Akers et al., 2014). The maze consisted of a round pool with a platform, and the platform was placed 1 cm under the water surface. The test contained two parts: the first one was place navigation trainings (5 days) and the other was spatial probe tests. Briefly, mice were put in the water from four quadrants of the maze every day, lasting for 5 days. The aim was to train the mice to locate the platform. When the mice failed to locate the platform in 60 s, they were guided to swim to the platform and remained there for 10 s of each trial. On the last day, the platform was taken out. Mice were put into the maze at the place opposite to the original location of the platform and were taken out after 60 s. The test parameters were recorded with an automated equipment (San Diego Instruments, San Diego, CA, USA).

### Rotarod Test

Briefly, mice ran on the accelerated rod three times a day, lasting for 3 days, with 2 days of training. The rod accelerated from 4 to 40 rpm in 300 s (Xin Ruan, Shanghai, China). Each mouse was allowed to rest for 30 min between experiments. The time of mice falling from the rod was recorded and the average was taken of the three tests.

### Open Field Test

In this experiment, the open field consisted of a white plastic box (45 × 45 × 45 cm). Locomotor activity was captured by a fixed camera and processed by a software (Xin Ruan, Shanghai, China). The animals were subjected to the open field test (OFT) for 5 min. The box was cleaned after each trial.

### Fecal Microbiota Analysis *via* 16S rRNA Sequencing

DNA of the fecal samples was extracted. Amplification of the V3 and V4 regions of the 16S rRNA gene was performed. Paired-end reads were generated on an Illumina MiSeq platform by following standard instructions. The tags consisted of high-quality paired-end reads and were clustered to operational taxonomic unit (OTU) at the level of 97% sequence similarity using the software USEARCH v7.0.1090. OTU taxonomy was divided on the basis of comparison with the Greengenes database (Fadrosh et al., 2014). According to the OTU abundance, Venn diagram was acquired by VennDiagram of software R (v3.1.1). The ACE, Chao1, Simpson, and Shannon parameters of α-diversity were analyzed. β-Diversity analysis was performed by partial least squares discriminant analysis (PLS-DA).

### Flow Cytometry

Periphery blood, spleens, and brains of mice treated with or without Pg were collected. Tissues of spleens and brains were ground and filtered through sterile cell filters. For blood and spleens, erythrocytes were lysed using RBC lysis buffer (CWBIO, Beijing, China) according to the instructions of the manufacturer and were then washed twice with PBS. A single-cell suspension of tissue was prepared. Anti-mouse CD16/32 monoclonal antibody (BioLegend) was used for blockage of Fc receptors. Dead cells were labeled with Zombie NIR Fixable Viability Kit (BioLegend). Cells were stimulated with Cell Activation Cocktail (BioLegend) and fixation/permeabilization was applied before intracellular staining. The antibodies were utilized for flow cytometry as follows: anti-mouse CD45 (clone 30-F11), anti-mouse CD11b (clone M1/70), anti-mouse CD3 (clone 145-2C11), anti-mouse CD4 (clone 145-2C11), anti-mouse CD8 (clone 53-6.7), and anti-mouse IFNγ (clone XMG1.2). All data were collected on a CytoFLEX (Beckman Coulter, USA) and analyzed with FlowJo software (version X, USA).

### Function Assessment of the Glymphatic Pathway

An *in vivo* two-photon microscope was used to assess the clearance function of the glymphatic pathway. The mice were anesthetized with pentobarbital (1%, 50 mg/kg). A slender cranial window was created about 3 mm in diameter using a stereotaxic device (RWD, Shenzhen, China). The view of the glymphatic pathway was observed by the two-photon microscope (Leica, Germany). Ten microliters of cerebrospinal fluid (CSF) tracer (FITC, Sigma-Aldrich, Germany) was injected into the cisterna magna with a duration of 10 min at a concentration of 1%. In order to make the blood vessels visible, rhodamine B dextran (Sigma, USA) was given by intravenous injection at a dosage of 0.2 ml per mouse. The operation was repeated at 5, 10, 15, 20, 25, 30, 45, and 60 min after the injection of the tracer. We analyzed the three-dimensional (3D) vectorized reconstruction of the distribution of the FITC tracer to observe its movement. For interstitial clearance, mean pixel intensities were also measured. All data acquisition was obtained by the Leica Lite software. The mean pixel intensities were measured in regions of interest throughout the time course and were normalized at the time of 5 min.

### TUNEL Staining

The TUNEL staining kit (Roche, USA) was utilized to assess the apoptotic neurocytes in the hippocampus and cortex. The procedures were conducted based on the instructions of the manufacturer. The number of TUNEL-positive nuclei was measured with ImageJ software. As a control, sections of brain tissue were operated by the same procedures in the absence of TdT enzyme.

### Immunofluorescence Staining

Sections of the brain were incubated with the following primary antibodies, including anti-IBA1 antibody (catalog number 019-19741, Wako, Japan), anti-Aβ1–42 (catalog number SIG-39142, BioLegend), and anti-GFAP (catalog number nG3893, Sigma-Aldrich), overnight at 4°C. The next day, the sections were incubated with secondary antibodies (catalog number 4408, 4413, Cell Signaling Technology) at room temperature for 1 h. The number of cells was calculated by two individuals using ImageJ software (version 1.46r, MD, USA).

### Data and Statistical Analyses

Two-way repeated measures ANOVA was used for the MWM measurements and the glymphatic system results, with Sidak’s test for multiple comparisons conducted. The difference between the two groups was evaluated by performing a *t*-test for normally distributed data and a non-parametric Mann–Whitney test for non-normal distribution. Data were expressed as means ± SEM, and *p*-value <0.05 was judged as significant difference (SPSS 19.0 software, USA; Prism 6, GraphPad, USA).

## Results

### *Porphyromonas gingivalis* Caused Behavioral Changes in Mice

**Figure 1:**
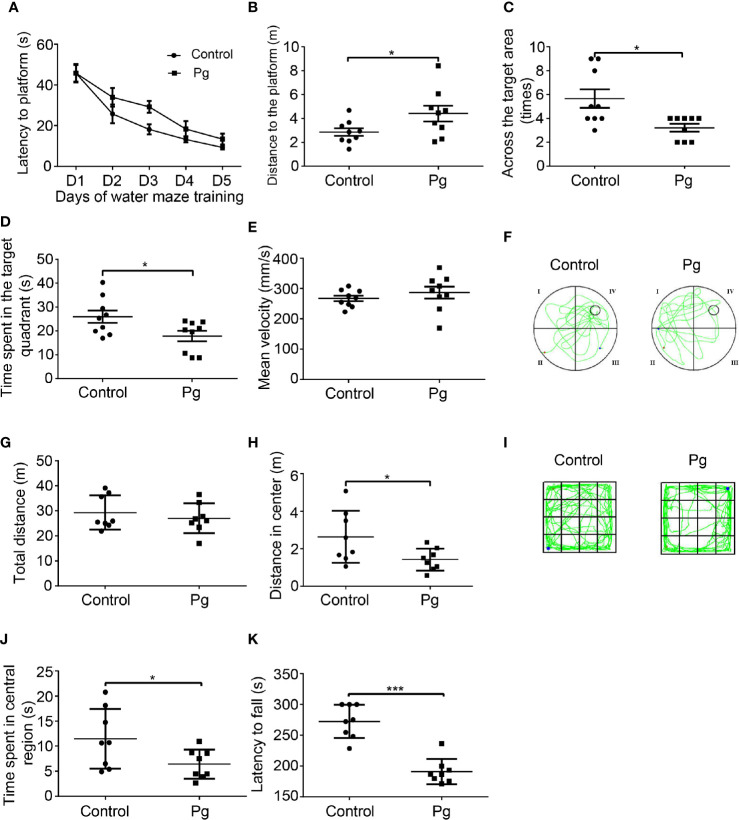
The effects of *Porphyromonas gingivalis* (Pg) on behavioral changes of mice. **(A)** Escape latencies in spatial acquisition trial of the Morris water maze (MWM). **(B)** The distance of mice to locate the platform on day 5. **(C)** The number of times the platform was crossed in the probe trial of the MWM. **(D)** Target quadrant movement time in the probe trial of the MWM. **(E)** Mean velocity of mice in the probe trial of the MWM. **(F)** Representative trajectories of each group in the MWM. **(G)** Total moving distance in the open field test (OFT); **(H)** distance in the central region of the OFT. **(I)** Representative trajectories of each group in the OFT. **(J)** Time spent in the central region of the OFT. **(K)** Latency to fall in the rotarod test. Each dot represents data from a mouse. Data were shown as means ± SEM. *p ≤ 0.05; ***p ≤ 0.001.

Pg administration had no negative effect on body weight (**Figure S1**). The MWM test was used to examine the learning and spatial memory of mice. The results of the 5-day training are shown in **Figure 1**. Pg-administered mice presented a longer escape latency on day 2 to day 5 (**Figure 1A**). Although the difference of latency was not significant, the longer latency during the training day somewhat reflected a slowed rate of spatial learning after Pg administration. Moreover, the distance that the Pg group traveled to locate the platform was significantly increased compared with the control group on day 5 (**Figure 1B**). The probe trial confirmed the presence of a spatial memory impairment in Pg-administered mice. The number of times crossing the target area was significantly decreased in the Pg group (3.22 ± 0.32) than that in the control group (5.67 ± 0.78, p < 0.05; **Figure 1C**). The time mice spent in the target quadrant was also significantly decreased in the Pg group (17.85 ± 2.18 s) than that in the control group (25.99 ± 2.63 s, p < 0.05; **Figure 1D**). Pg-treated mice did not recall the location of the platform and explored other quadrants (**Figure 1F**). There was no significant difference of the swimming speeds between the two groups (**Figure 1E**). Taken together, our results demonstrated that Pg worsens the function of spatial cognition of mice.

To evaluate the general locomotor activity of the mice and their willingness to explore, the OFT was carried out. Indeed, Pg-administered mice spent less time in the central region of the box and showed little interest in exploring when compared with the control mice (**Figure 1J**). Moreover, Pg-treated mice showed a significant reduction in the central distance traveled (1.42 ± 0.21 m), when compared with the control group (2.63 ± 0.49 m, p < 0.05; **Figure 1H**). There was no significant difference in total moving distance between the control and Pg group (**Figure 1G**). The representative trajectories of both groups are shown in **Figure 1I**.

To assess motor function and fatigue level, performance on the accelerating rotarod was recorded. The latency of falling from the rotarod of both groups was 272.30 ± 9.57 s (control) and 191.00 ± 7.26 s (Pg). Administration with Pg significantly decreased the riding time by 29.9% compared with that of the control group (*p* < 0.001; **Figure 1K**).

### Oral Administration of Pg Altered Gut Microbiota Composition

**Figure 2:**
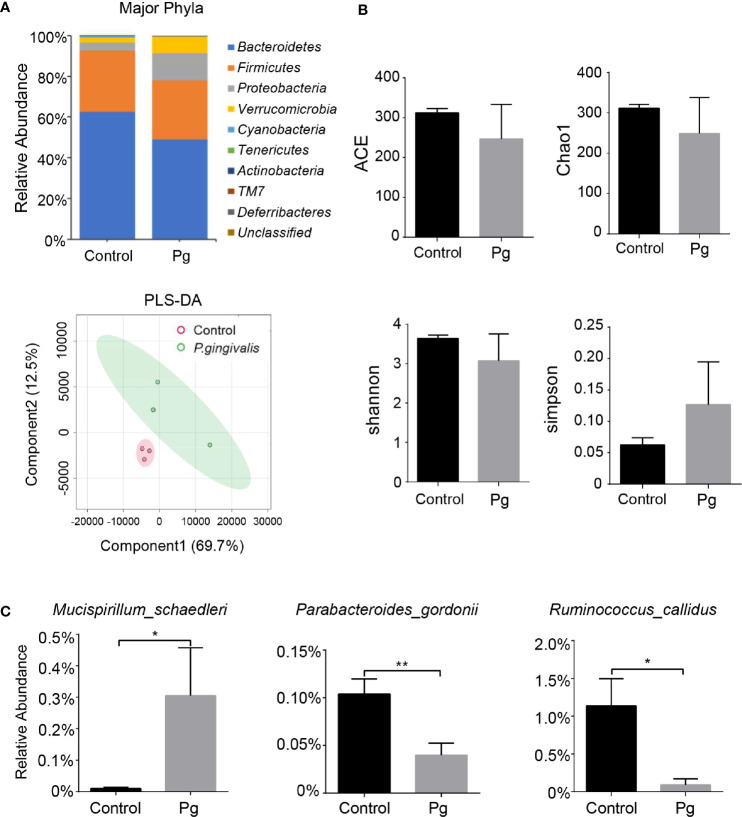
Influence of oral gavage with Pg on the composition of gut microbiota. Mice were subjected to oral gavage with either 10^9^ CFU of Pg or CMC three times a week for 4 weeks. Stool samples were used for 16S rRNA sequencing. **(A)** At the phyla level, the relative abundance of bacteria in the Pg-administered and control groups. **(B)** Alpha- and beta-diversity of the gut microbiota in the Pg-administered and control groups. **(C)** The significant differences in relative abundance of species between the two groups. *n* = 3, data were shown as means ± SEM. *p ≤ 0.05; **p ≤ 0.01.

To evaluate the influence of Pg gavage on gut microbiota, feces were analyzed for their microbiota composition. The composition ratio of the gut microbiota changed (**Figure 2A** and **Figures S2A, B**). The number of shared OTUs in both groups was 334 as shown in the Venn diagrams, and the unique OTUs of the two groups were 60 in the control group and 52 in the Pg group, respectively (**Figure S2C**). The α-diversity parameters, including ACE, Chao1, Shannon, and Simpson, were analyzed. None of the parameters showed a significant change by repeated administration of Pg (**Figure 2B**). The result of PLS-DA analysis displayed that the samples could be divided into two parts. This demonstrated that the gut microbiota compositions were different between the Pg-administered and control groups (**Figure 2B**). At the phylum level, the proportion of *Tenericutes* was significantly increased in Pg-treated mice than in control ones, and the proportion of *Actinobacteria* was slightly decreased (**Figure S2D**). At the class level, the proportion of *Coriobacteriia* was significantly decreased in the Pg group than in the control group, while the proportion of *Mollicutes* was significantly increased (**Figure S2D**). At the order level, the proportion of *Coriobacteriales* was significantly decreased in Pg-treated mice than in control ones (**Figure S2D**). At the family level, the proportions of *Clostridiaceae*, *Coriobacteriaceae*, and *Prevotellaceae* were significantly decreased in the Pg group, and that of *S24-7* was slightly decreased. At the genus level, the proportion of *Prevotella* was significantly decreased in the Pg group than in the control group (**Figure S2D**). At the species level, the proportions of *Parabacteroides gordonii* and *Ruminococcus callidus* were significantly decreased in Pg-treated mice than in control ones, whereas the proportion of *Mucispirillum schaedleri* was significantly increased (**Figure 2C**). Besides, the ileum of the Pg group showed partial intestinal gland destruction and inflammatory cell infiltration. These results demonstrated that gut microbiota dysbiosis caused by Pg administration can induce intestinal inflammatory response. However, there was no histopathologic change in the colon (**Figure S3**).

### Pg Changed the Immune Environment After Gut Microbiota Dysbiosis

**Figure 3:**
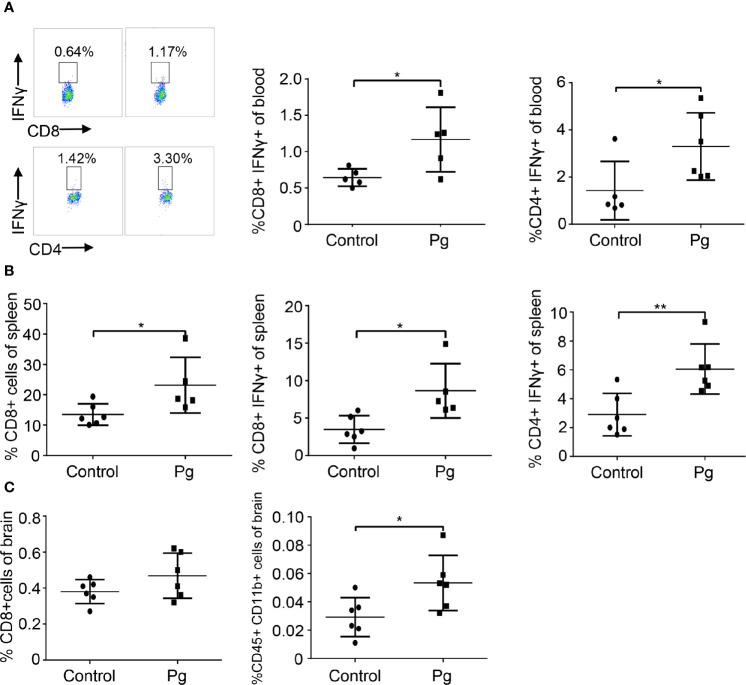
Pg-administered mice show changes in proportions of periphery lymphocytes and brain-infiltrating immune cell subsets. (A) Flow cytometry is used to analyze the composition of blood cells in the control and Pg-administered group. Numbers represent the percentage of the target cell group in blood cells. (B) Flow cytometry is used to analyze the composition of spleen cells. (C) Flow cytometry is used to analyze the composition of brain-infiltrating immune cells. Each dot represents data from a mouse. Data were shown as means ± SEM. *p ≤ 0.05; **p ≤ 0.01.

To address whether Pg contributed to brain disorders by affecting the immune pathway of the GBA, we detected immune cells from the blood, spleen, and brain of mice. The proportions of CD4^+^IFNγ^+^ T cells and CD8^+^IFNγ^+^ T cells were increased in the blood and spleen of mice with Pg gavage compared with those of the control mice (**Figures 3A, B**). The proportion of CD8^+^ T cells of the spleen was significantly increased in the Pg group, while that of the brain was slightly increased. However, the proportion of CD45^+^CD11b^+^ myeloid cells was significantly increased in the brain of the Pg group (**Figure 3C**).

### Dysfunction of the Glymphatic System

**Figure 4:**
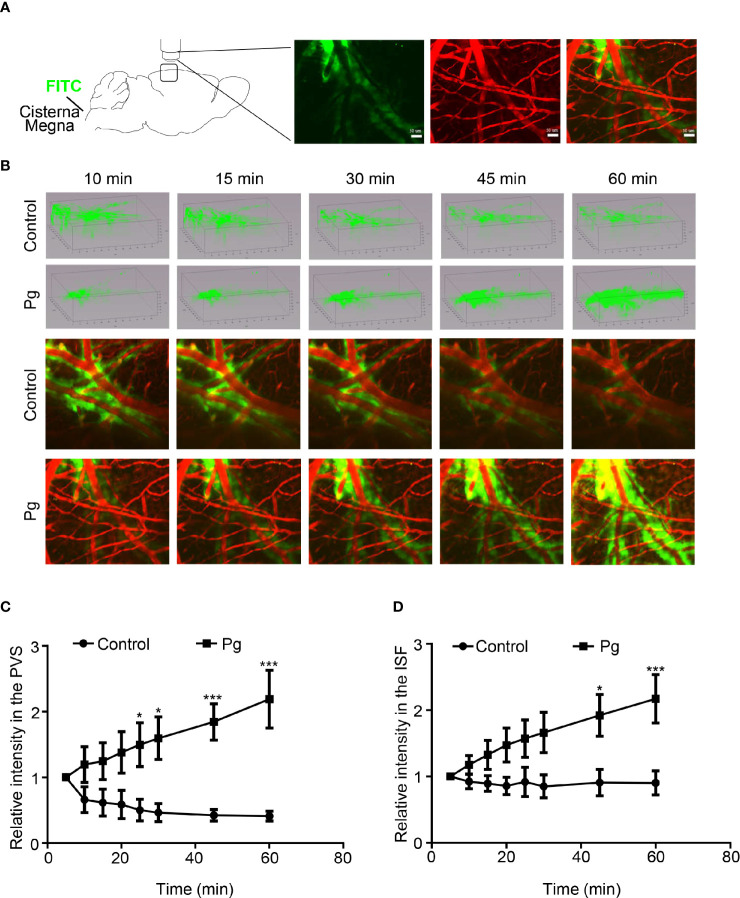
Clearance function of the glymphatic system, including inflow of the cerebrospinal fluid (CSF) through PVS–ISF exchange and the outflow of ISF drainage. **(A)** Diagram representing the two-photon microscopic image of the CSF tracer into the cisterna magna. **(B)** Three-dimensional images of the distribution of the CSF tracer in the Pg and control groups. Representative picture of the CSF tracer entering the brain parenchyma along the PVS. **(C)** Comparison of the relative fluorescence intensity in the PVS. **(D)** Comparison of the relative fluorescence intensity in the ISF between the control and Pg group. *n* = 4, data were shown as means ± SEM, *p ≤ 0.05; ***p ≤ 0.001. Scale bar, 50 μm.

The clearance function of the glymphatic system was measured in mice. The CSF tracer was given to the cisterna magna by infusion and the blood was visualized by intravenous injection of rhodamine B dextran (**Figure 4A**). The CSF tracer ran to the cortex along the permeating arterioles and went into ISF of the parenchyma through PVS. The CSF tracer in the PVS of the permeating arteries was analyzed 100 μm under the surface of the cortex (**Figure 4B**). In control mice, the measurement of the CSF tracer in pixel intensity at 5 min was set as a baseline. The relative pixel intensity along the PVS in control mice was gradually decreased over time. In contrast, the CSF tracer was accumulated along the PVS in Pg-treated mice, and the relative pixel intensity was significantly increased at 25, 30, 45, and 60 min (**Figure 4C**). These results indicated that oral gavage with Pg decreased the CSF–ISF exchange of the brain. We also analyzed the pixel intensity of the CSF tracer in brain parenchyma. In control mice, the relative pixel intensity stayed nearly at the same level during the testing time. However, in the Pg group, the relative pixel intensity was significantly increased at 45 and 60 min (**Figure 4D**). It indicated that Pg of oral origin impaired the ISF drainage of the brain.

### Pg Aggravated Neuroinflammation in the Brain

**Figure 5:**
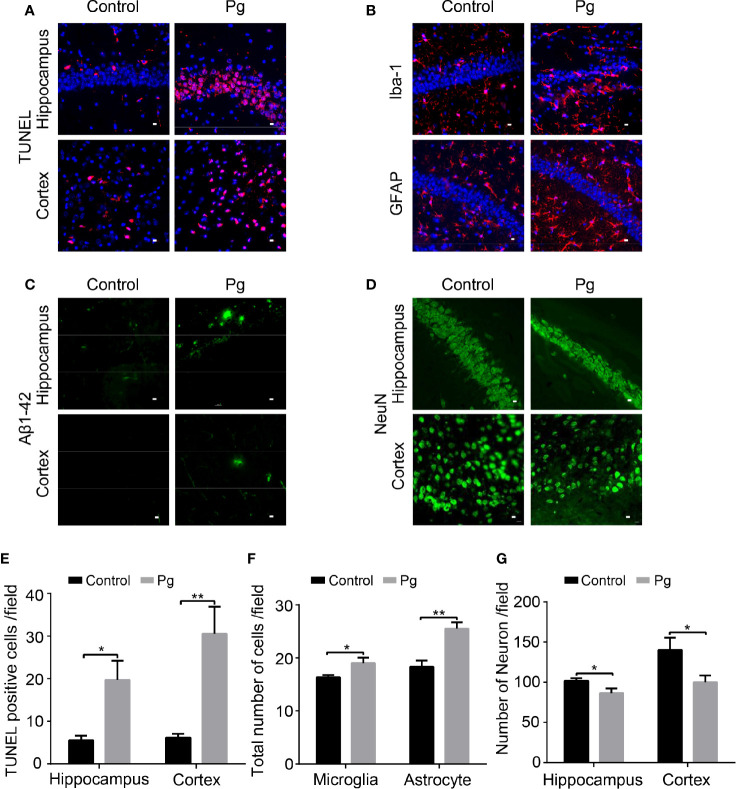
Immunohistochemical staining of the hippocampus and cortex. **(A)** Representative image presenting TUNEL-positive cells in the hippocampus and cortex. **(B)** Representative image presenting Iba-1 and GFAP-immunopositive cells in the hippocampus. **(C)** Representative section showing amyloid plaque in the hippocampus and cortex. **(D)** Representative image presenting NeuN-positive cells in the hippocampus and cortex. **(E)** Comparison of the difference in the number of TUNEL-positive cells. **(F)** Comparison of the difference in the number of GFAP and Iba-1-positive cells in the hippocampus. **(G)** Comparison of the difference in the number of NeuN-positive cells in the hippocampus and cortex. *n* = 6, data were shown as means ± SEM. *p ≤ 0.05; **p ≤ 0.01. Scale bar, 10 μm.

Overactivation of neuroinflammation is reported to be associated with neurodegeneration in AD (Heneka et al., 2015). We conducted immunofluorescence staining to explore the histopathologic changes induced by Pg. We gauged and compared the positive cells of TUNEL, neurons (NeuN), microglia (Iba-1), and astrocytes (GFAP) in the cortex and hippocampus regions of mice in different groups. The dysbiosis of gut microbiota and infiltration of immune cells can promote inflammatory activation of glial cells. Pg increased over 16.35% of the number of microglia and 39.12% of the number of astrocytes in the hippocampus region than those in the control group (**Figures 5B, F**). Neuroinflammation may induce apoptosis of neurocytes. Pg increased the number of TUNEL-positive cells in the hippocampus and cortex regions than those in control mice (**Figures 5A, E**). Moreover, the number of neurons in the hippocampus and cortex regions was significantly decreased in Pg-administered mice (**Figures 5D, G**). In addition, amyloid plaque appeared in those two brain regions of the Pg group (**Figure 5C**).

## Discussion

In this study, Pg of oral origin induced dysbiosis of gut microbiota. Microbiota was a potent regulator of host immune responses, and the T lymphocytes and myeloid cells were increased in the peripheral and CNS, respectively. Changes of the CNS immune microenvironment exacerbated the neuroinflammation. Furthermore, the solute clearance function of the glymphatic system was reduced. Aβ plaques were shown in the brain and cognitive function was prominently impaired in mice that were subjected to oral gavage with Pg. Hence, this study provides further evidence for Pg exacerbating neuroinflammation, impairing glymphatic function, and ultimately leading to a decline in cognitive function by disturbing the GBA.

In order to mimic clinical conditions, the dose of Pg given to mice was calculated according to the quantity of microbial load and saliva swallowed by a patient with periodontitis. The time of Pg administration was based on how long periodontitis can be induced by bacteria in mice (Boyer et al., 2020). Pg can act either directly or indirectly on the brain. On the one hand, Pg is detected in the brain of AD patients. Pg in the brain may induce neuroinflammation by secreting gingipain and results in the deposition of amyloid protein (Dominy et al., 2019). In addition to the direct action, Pg can act indirectly on neuroinflammation through gut dysbiosis. Gut dysbiosis causes an increase in inflammatory T and B lymphocytes and a subsequent systemic inflammation, thereby inducing neuroinflammation (Suganya and Koo, 2020; Carlessi et al., 2021). Second, gut dysbiosis decreases the production of short-chain fatty acids (SCFAs), which is related to inflammatory responses (Lach et al., 2018; Gudi et al., 2020). Third, gut dysbiosis exacerbates neuroinflammation by transmitting gut-derived Aβ to the brain through the vagus nerve (Chen et al., 2021).

The oral cavity–gut–multiorgan axis has been recently proposed to link periodontitis to systemic diseases (Kato et al., 2018). Indeed, Pg can be detected in the fecal collections from patients with colorectal cancers (Wang et al., 2021). It is reported that during periodontal diseases, extension of the oral microbiota can promote inflammatory bowel disease by ectopic gut colonization (Kitamoto et al., 2020). An outgrowth of potentially pathogenic bacteria and a decrease of beneficial bacteria may result in pathological changes of gut tissue. In the present study, we found inflammatory cells infiltrated in the ileum tissue and some destruction of the intestinal glands. Besides, at the species level, the proportions of *M. schaedleri*, *P. gordonii*, and *R. callidus* were changed in the Pg group. *Mucispirillum schaedleri* is detected in a variety of mammals, and it is known to have low relative abundance of the intestinal microbiota in murine feces (Herp et al., 2021). An increasing number of certain bacterial species have been associated with inflammatory conditions in the gut (Selmin et al., 2021). *Parabacteroides gordonii* is one of the several gut bacteria with anti-inflammatory attributes (Abais-Battad et al., 2021). *Ruminococcus callidus* which produces SCFAs is considered as a biomarker for improving health (Sanchez-Tapia et al., 2020). Moreover, the relative abundances of both bacteria were decreased in the Pg group. The changes of the above three bacterial species were associated with inflammatory pathology of the ileum. In addition, Pg administration has been documented to modulate gut microbiota and gut immune system. Pg could shift the proportion of T lymphocytes to inflammatory T cells in mesenteric lymph node by disturbing the gut microbiota, and the level of proinflammatory cytokine in sera is also increased. Besides, oral gavage with Pg can impair the barrier function of intestinal epithelium and decrease the expression of tight junction protein ZO-1 (Kato et al., 2018; Feng Y. K. et al., 2020; Tsuzuno et al., 2021). Further studies are urgently needed to clarify the pathway how Pg causes pathologic and microbiological changes of the gut.

In accordance with a previous study, gut microbiota played an important role in behavior (Oh et al., 2015), and animals in the Pg group were more prone to fatigue in the rotarod test. In addition, Pg-treated mice showed little interest in exploration, indicating anxiety-like behavior. These results indicated that dysbiosis of gut microbiota caused by Pg administration could disturb the brain function. The gut microbiota is critical for the maturation and proper function of microglia, while dysbiosis of gut microbiota induces neuroinflammation (Erny et al., 2015). AD mice exhibit altered gut microbiota compositions, which is positively correlated with enhanced astrogliosis and microgliosis in the brain (Shukla et al., 2021). Conversely, rescuing the dysbiosis of gut microbiota can suppress the microglia activation and downregulate the production of proinflammatory cytokines (Shen et al., 2020). Consistently, we found that Pg of oral origin could induce astrogliosis and microgliosis in the brain. Dysbiosis of gut microbiota has been strongly involved in the development of neurodegenerative disorders through modulating the GBA (Stefano et al., 2018; Cryan et al., 2020; Zhu et al., 2020). The gut microbiota can modify immune cells and promote the production of proinflammatory cytokines (Hegazy et al., 2017; McCoy et al., 2017; Zhao and Elson, 2018). Thereafter, immune cells along with inflammatory mediators may infiltrate the brain (Campos-Acuna et al., 2019). In this study, the proportions of CD4^+^IFNγ^+^ T lymphocytes and CD8^+^IFNγ^+^ T cells were increased in the blood and spleen of Pg-treated mice, and the peripheral Th1 (CD4^+^IFNγ^+^) cells are associated with M1 microglia activation and contribute to neuroinflammation (Wang et al., 2019). A study limitation is that we did not examine the levels of IFNγ in the sera, gut, and brain. Furthermore, we observed that Pg promoted the infiltration of myeloid cells (CD45^+^CD11b^+^) in the brain. The mechanisms by which myeloid cells activate neuroinflammation are still under examination. Further studies are needed to clarify whether removing Pg can rescue the damage of the brain.

In addition, the gut microbiome imbalance and associated neuroinflammation may disrupt CNS fluid flow, which leads to the breakdown of the glymphatic system (Rustenhoven et al., 2021). The recently discovered glymphatic system in brain parenchyma and the meningeal lymphatics are recognized as vital pathways for clearance of toxic solutes from the brain (Da Mesquita et al., 2021). The dysfunction of the glymphatic CSF–ISF exchange has been implicated in the initiation and progression of AD and Parkinson’s disease (Wood, 2021). In our study, the clearance rate of both the PVS and ISF bulk flow in the Pg group was much slower than that in the control group. This accelerated the accumulation of interstitial waste from the brain parenchyma. In correspondence, the deposition of Aβ plaque in the brain was observed in the Pg group. The bidirectional interaction between Aβ deposition and neuroinflammation resulted in the apoptosis of neurocytes and then impaired cognitive function in the Pg group. It is reported that inhibition of Pg-induced neuroinflammation can decrease the deposition of Aβ (Dominy et al., 2019; Liu et al., 2020).

The glymphatic system mainly consists of astrocytes where aquaporin-4 (AQP4) water channels locate (Mestre et al., 2018). Astrocytes are a group of glial cells in abundance in the CNS that have significant homeostatic maintenance and disease-promoting function. Immune cells that are licensed by the gut microbiota can modulate the function of astrocytes (Sanmarco et al., 2021). Moreover, in this study, reactive proliferation of astrocytes was observed in the Pg group, which is related with the dysfunction of the glymphatic system. Furthermore, the localization of AQP4 is highly polarized to perivascular endfeet of astrocytes that facilitate the periarterial CSF influx and the perivenous ISF clearance pathways (Feng W. et al., 2020; Hablitz et al., 2020). Dysfunction of astrocytes, including reactive astrogliosis, causes abnormal production and position of AQP4, which disturbs the clearance function of adverse solutes in the brain in turn. Future work will address whether Pg can modify the polarization of AQP4 water channel by interacting with the GBA.

## Conclusions

Our results indicate that periodontal pathogen Pg induces cognitive decline, accompanied by gut microbiota dysbiosis, neuroinflammation, and glymphatic system impairment. In conclusion, the present study suggests a potential role of Pg-induced dysfunction of the GBA in the pathophysiology of cognitive impairment.

## Data Availability Statement

The original contributions presented in the study are publicly available in Figshare: DOI: 10.6084/m9.figshare.17081648.

## Ethics Statement

The animal study was reviewed and approved by the Institutional Animal Care and Use Committee, Sun Yat-Sen University.

## Author Contributions

LC, ZP, and WT designed the studies. LC and XC performed the experiments, analyzed the data, and wrote the manuscript. LL, TY, JS, YF, and FL performed some of the experiments. ZP and WT supervised the project and revised and approved the final version of the manuscript. All authors contributed to the article and approved the submitted version.

## Funding

This study was supported by grants from the National Natural Science Foundation of China (No. 82071255, No. 81873751); National Key Research and Development Program of China, Stem Cell and Translational Research (No. 2017YFA0105104); Guangdong Provincial Science and Technology Plan Project (No. 2016B030230002); Southern China International Cooperation Base for Early Intervention and Functional Rehabilitation of Neurological Diseases (No. 2015B050501003); Guangdong Provincial Engineering Center for Major Neurological Disease Treatment; Guangdong Provincial Translational Medicine Innovation Platform for Diagnosis and Treatment of Major Neurological Disease; and Guangdong Provincial Clinical Research Center for Neurological Diseases.

## Conflict of Interest

The authors declare that the research was conducted in the absence of any commercial or financial relationships that could be construed as a potential conflict of interest.

## Publisher’s Note

All claims expressed in this article are solely those of the authors and do not necessarily represent those of their affiliated organizations, or those of the publisher, the editors and the reviewers. Any product that may be evaluated in this article, or claim that may be made by its manufacturer, is not guaranteed or endorsed by the publisher.

## Supplementary Material

Supplementary Figure 1 Physiological measures of body weight after the first Pg administration.

Supplementary Figure 2
**(A)** The composition of bacteria at the Class level between the Pg-administered and control group. **(B)** The composition of bacteria at the Genus level between the Pg-administered and control group. **(C)** Venn diagrams are used to show the number of common or unique OTUs between Pg and control group. Each circle represents the number of OTUs for each group. The overlapping area represents the shared OTUs in both groups. **(D)** Bar plots for phylum, class, order, family and genus that showed differences in the Pg-administered and control group. n=3, data were shown as means ± SEM. **p* ≤ 0.05; ***p* ≤ 0.01.

Supplementary Figure 3 Histological aspects of ileum and colon. **(A)** Representative histological section of ileum. Rectangular box represents the gland destruction in the gut and inflammatory cells infiltration. **(B)** Representative histological section of colon. Scale bar, 200 μm.
