# Supplementary figures and images for: Porphyromonas gingivalis-Induced Cognitive Impairment Is Associated With Gut Dysbiosis, Neuroinflammation, and Glymphatic Dysfunction
Source: Front Cell Infect Microbiol. 2021 Dec 1;11:755925. doi: 10.3389/fcimb.2021.755925 (PMC8672439; doi:10.3389/fcimb.2021.755925)

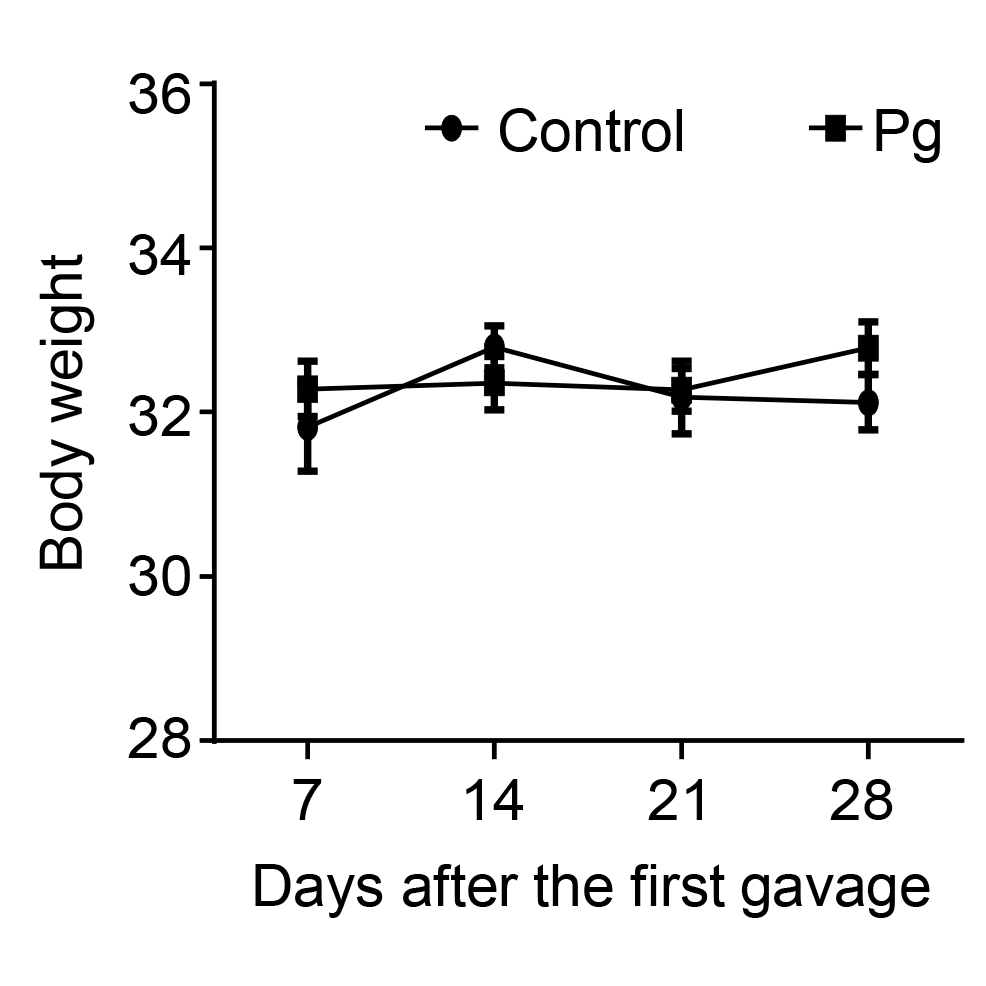

Supplement: Supplementary Figure 1 — Physiological measures of body weight after the first Pg administration. [file Image_1.tif]

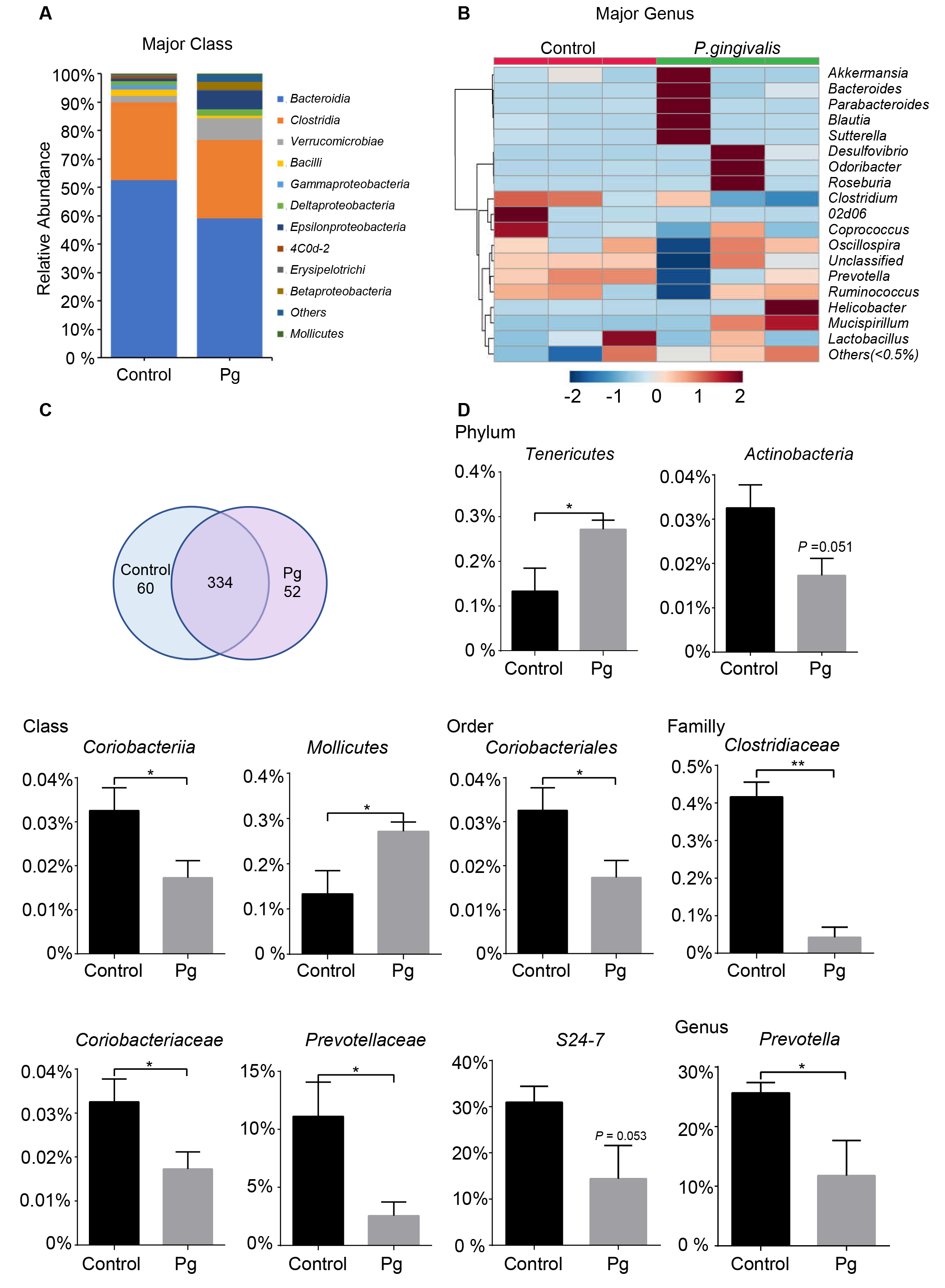

Supplement: Supplementary Figure 2 — (A) The composition of bacteria at the Class level between the Pg-administered and control group. (B) The composition of bacteria at the Genus level between the Pg-administered and control group. (C) Venn diagrams are used to show the number of common or unique OTUs between Pg and control group. Each circle represents the number of OTUs for each group. The overlapping area represents the shared OTUs in both groups. (D) Bar plots for phylum, class, order, family and genus that showed differences in the Pg-administered and control group. n=3, data were shown as means ± SEM. *p ≤ 0.05; **p ≤ 0.01. [file Image_2.tif]

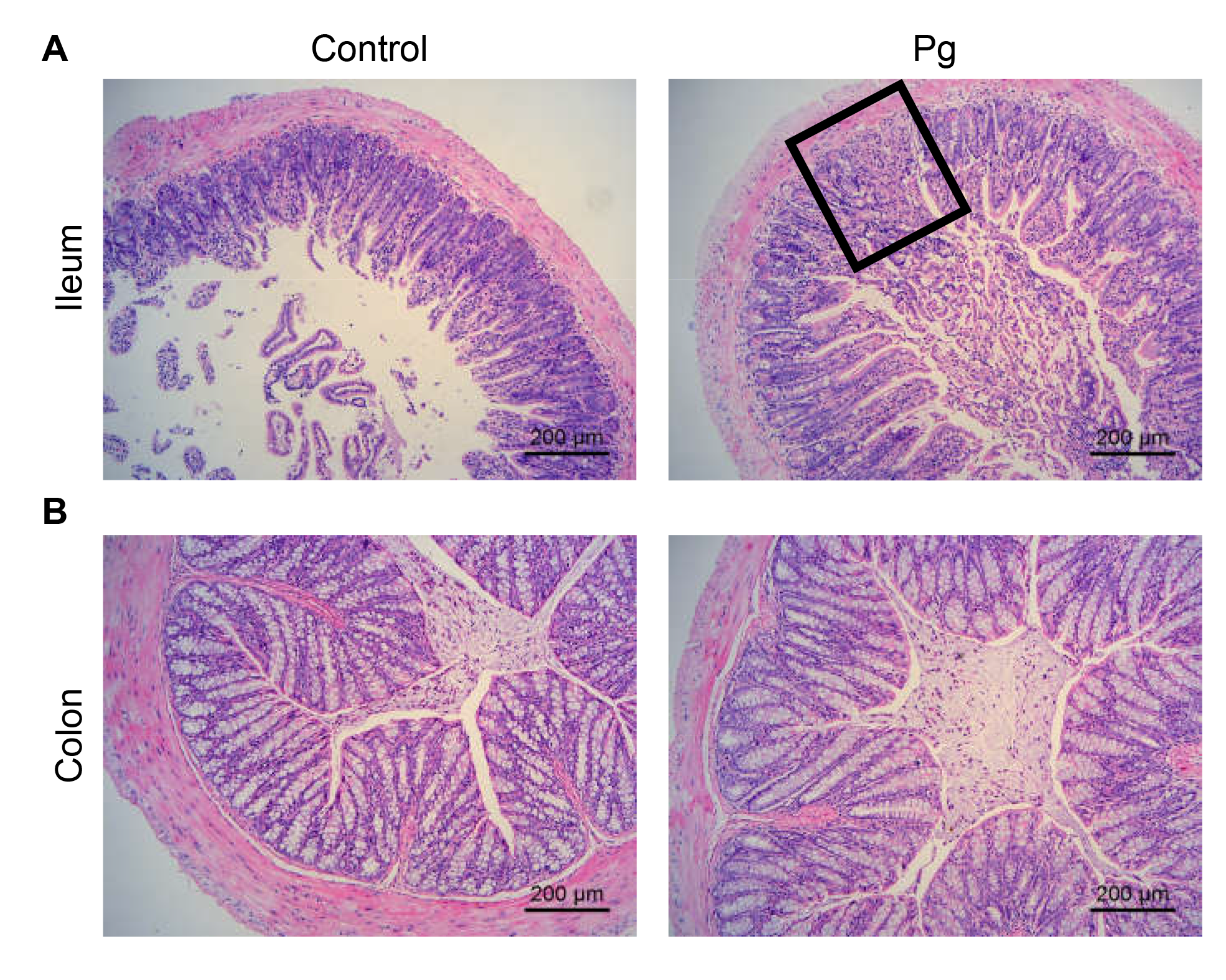

Supplement: Supplementary Figure 3 — Histological aspects of ileum and colon. (A) Representative histological section of ileum. Rectangular box represents the gland destruction in the gut and inflammatory cells infiltration. (B) Representative histological section of colon. Scale bar, 200 μm. [file Image_3.tif]
