# Supplementary material for: Porphyromonas gingivalis-Induced Cognitive Impairment Is Associated With Gut Dysbiosis, Neuroinflammation, and Glymphatic Dysfunction
Source: Front Cell Infect Microbiol. 2021 Dec 1;11:755925. doi: 10.3389/fcimb.2021.755925 (PMC8672439; doi:10.3389/fcimb.2021.755925)
Supplement: Supplementary file 5 [file Table_2.docx]

CD45+CD11b cells: <https://www.jianguoyun.com/p/DTgMR-4QqLDeCRjAgoYE>

Immunofluorescence staining: <https://www.jianguoyun.com/p/DWD-ejUQyrveCRiRkoYE>

T cells: <https://www.jianguoyun.com/p/DfIc4wEQsrLeCRi1q4cE>
